# Archives of Surgery

**Published:** 1890-09

**Authors:** 


*Archives of Surgery.* By JONATHAN HUTCHINSON, LL.D., F.R.S. 1889-90. London: J. & A. CHURCHILL.

This quarterly periodical, conducted, and written almost entirely, by Mr. Jonathan Hutchinson, has recently been added to our list of Exchanges. It is a most valuable publication, characterised by Mr. Hutchinson's well-known breadth of view and clearness of diction. It is not confined to an exposition of surgery only; but, in addition to a masterly philosophical dissertation here and there, includes many other topics on which Mr. Hutchinson is a recognised authority. It is well printed and admirably illustrated; and in conjunction with Dr. Richardson's *Asclepiad* and Dr. Byrom Bramwell's *Studies in Clinical Medicine,* should act as an incentive to others of wide experience and untiring industry to make common property of their stores of knowledge and of clinical material.

We hope Mr. Hutchinson will not limit himself to the three years' issue at which he hints in his prefatory note.

